# Landscape Encodings Enhance Optimization

**DOI:** 10.1371/journal.pone.0034780

**Published:** 2012-04-09

**Authors:** Konstantin Klemm, Anita Mehta, Peter F. Stadler

**Affiliations:** 1 Bioinformatics Group, Department of Computer Science, and Interdisciplinary Center for Bioinformatics, University of Leipzig, Leipzig, Germany; 2 Theory Department, S N Bose National Centre for Basic Sciences, Calcutta, India; 3 Max Planck Institute for Mathematics in the Science, Leipzig, Germany; 4 Fraunhofer Institut für Zelltherapie und Immunologie, Leipzig, Germany; 5 Department of Theoretical Chemistry, University of Vienna, Vienna, Austria; 6 Santa Fe Institute, Santa Fe, New Mexico, United States of America; Universitat Rovira i Virgili, Spain

## Abstract

Hard combinatorial optimization problems deal with the search for the minimum cost solutions (ground states) of discrete systems under strong constraints. A transformation of state variables may enhance computational tractability. It has been argued that these state encodings are to be chosen invertible to retain the original size of the state space. Here we show how redundant non-invertible encodings enhance optimization by enriching the density of low-energy states. In addition, smooth landscapes may be established on encoded state spaces to guide local search dynamics towards the ground state.

## Introduction

Complex systems in our world are often *computationally* complex as well. In particular, the class of NP-complete problems [1], for which no fast solvers are known, encompasses not only a wide variety of well-known combinatorial optimization problems from the Travelling Salesman Problem to graph coloring, but also includes a rich diversity of applications in the natural sciences ranging from genetic networks [2] through protein folding [3] to spin glasses [4]–[7]. In such cases, heuristic optimization – where the goal is to find the best solution that is reachable within an allocated time – is widely accepted as being a more fruitful avenue of research than attempting to find an exact, globally optimal, solution. This view is motivated at least in part by the realization that in physical and biological systems, there are severe constraints on the type of algorithms that can be naturally implemented as dynamical processes. Typically, thus, we have to deal with local search algorithms. Simulated annealing [8], genetic and evolutionary algorithms [9], as well as genetic programming [10] are the most prominent representatives of this type. Their common principle is the generation of variation by thermal or mutational noise, and the subsequent selection of variants that are advantageous in terms of energy or fitness [11].

The performance of such local search heuristics naturally depends on the structure of the search space, which, in turn, depends on two ingredients: (1) the encoding of the configurations and (2) a move set. Many combinatorial optimization problems as well as their counterparts in statistical physics, such as spin glass models, admit a natural encoding that is (essentially) free of redundancy. In the evolutionary computation literature this “direct encoding” is often referred to as the “phenotype space”, 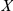. The complexity of optimizing a cost function 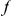 over 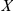 is determined already at this level. For simplicity, we call 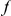 energy and refer to its global minima as ground states. In evolutionary computation, one often uses an additional encoding 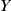, called the “genotype space” on which search operators, such as mutation and cross-over, are defined more conveniently [12], [13]. The genotype-phenotype relation is determined by a map 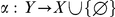, where 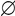 represents phenotypic configurations that do not occur in the original problem, *i.e.* non-feasible solutions. For example, the tours of a Traveling Salesman Problem (TSP) [14] are directly encoded as permutations describing the order of the cities along the tour. A frequently used encoding as binary strings represents every connection between cities as a bit that can be present or absent in a tour; of course, most binary strings do not refer to valid tours in this picture.

The move set (or more generally the search operators [15]) define a notion of locality on 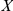. Here we are interested only in mutation-based search, where for each 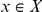 there is a set of neighbors 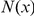 that is reachable in a single step. Such neighboring configurations are said to be *neutral* if they have the same fitness. Detailed investigations of fitness landscapes arising from molecular biology have led to the conclusion that high degrees of neutrality *can* facilitate optimization [11], [16]. More precisely, when populations are trapped in a metastable phenotypic state, they are most likely to escape by crossing an entropy barrier, along long neutral paths that traverse large portions of genotype space [17].

In contrast, some authors advocate to use “synonymous encodings” for the design of evolutionary algorithms, where genotypes mapping to the same phenotype 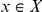 are very similar, i.e., 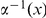 forms a local “cluster” in 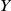, see e.g. [13], [18], [19]. This picture is incompatible with the advantages of extensive neutral paths observed in biologically inspired landscape models [16], [20] and in genetic programming [21], [22]. An empirical study [23], furthermore, shows that the introduction of arbitrary redundancy (by means of random Boolean network mapping) does not increase the performance of mutation-based search. This observation can be understood in terms of a random graph model of neutral networks, in which only very high levels of randomized redundancy result in the emergence of neutral paths [24].

An important feature that appears to have been overlooked in most recent literature is that the redundancy of 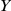 with respect to 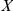 need not be homogeneous [12]. Inhomogeneous redundancy implies that the size of the preimage 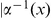 may depend on 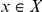. If 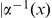 is anti-correlated with the energy 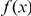, then the encoding 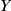 enables the preferential sampling of low-energy states in 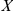. Thus even a random selection of a state yields lower energy when performed in 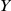 than in 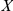. Here we demonstrate this *enrichment* of low energy states for three established combinatorial optimization problems and suitably chosen encodings. The necessary formal aspects of energy landscapes and their encodings are outlined in the Methods section. We formalize and measure enrichment in terms of densities of states on 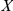 and 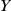, see *Methods* for a formal treatment. We illustrate the effects of encoding by comparing performance of optimization heuristics on the direct and encoded landscapes.

## Results and Discussion

### Number Partitioning

The first optimization problem we consider is the number partitioning problem (NPP) [1]: this asks if one can divide 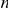 positive numbers 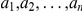 into two subsets such that the sum of elements in the first subset is the same as the sum over elements in the other subset. The energy is defined as the deviation from equal sums in the two subsets, i.e.,
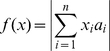
(1)where the two choices 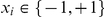 correspond to assignment to the first or to the second subset, respectively. The flipping of one of the spin variables 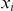 is used as a move set, so that the NPP landscape is built on a hypercube. The NPP shows a phase transition between an easy and a hard phase. We consider here only instances that are hard in practice, i.e., where the coefficients 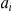 have a sufficiently large number of digits [25].

The so-called *prepartitioning* encoding [26] of the NPP is based on the differencing heuristic by Karmakar and Karp [27]. Departing from an NPP instance 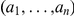, the heuristic removes the largest number, say 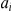, and the second largest 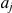 and replaces them by their difference 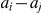. This reduces the problem size from 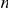 to 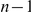. After iterating this differencing step 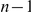 times, the single remaining number is an upper bound for – and in many cases a good approximation to – the global minimum energy. The minimizing configuration itself is obtained by keeping track of the items chosen for differencing. Replacing 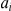 and 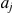 by their difference amounts to putting 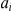 and 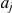 into different subsets, i.e. 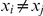.

The prepartitioning encoding is obtained by modifying the initial condition of the heuristic. Each number 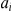 is assigned a class 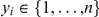. A new NPP instance 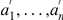 is generated by adding up all numbers 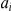 in the same class 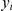 into a single number 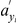. After removing zeros from 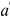, the differencing heuristic is applied to 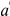. In short: 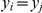 imposes the constraint 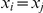. Running the heuristic under this constraint, the resulting configuration 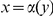 is unique up to flipping all spins in 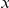. The so defined mapping 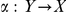 is surjective because for each 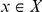, 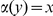 for 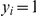 if 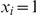 and 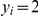 otherwise. Two encodings 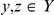 are neighbors if they differ at exactly one index 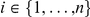. This encoding is the one whose performance we will compare with the direct encoding later.

### Traveling Salesman

Our next optimization problem, the Traveling Salesman Problem, (TSP) is another classical NP-hard optimization problem [1]. Given a set of 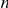 vertices (cities, locations) 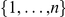 and a symmetric matrix of distances or travel costs 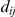, the task is to find a permutation (tour) 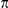 that minimizes the total travel cost
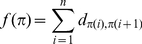
(2)where indices are interpreted modulo 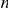. Here, the states of the landscape are the permutations of 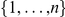, 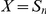. Two permutations 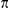 and 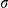 are adjacent, 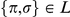, if they differ by one *reversal*. This means that there are indices 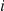 and 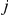 with 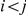 such that 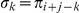 for 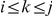 and 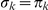 otherwise.

Similar to the NPP case, an encoding configuration 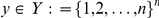 acts as a constraint. A tour 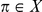 fulfills 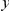 if for all cities 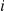 and 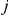, 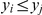 implies 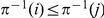. Thus 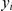 is the relative position of city 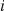 in the tour since it must come after all cities 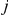 with 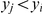. All cities with the same 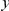-value appear in a single section along the tour. If there are no two cities with the same 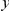-value then 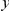 itself is a permutation and there is a unique 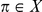 obeying 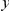, namely 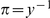.

Among the tours compatible with the constraint, a selection is made with the greedy algorithm. It constructs a tour by iteratively fixing adjacencies of cities. Starting from an empty set of adjacencies, we attempt to include an adjacency 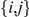 at each step. If the resulting set of adjacencies is still a subset of a valid tour obeying the constraint, the addition is accepted, otherwise 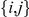 is discarded. The step is iterated, proposing each 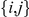 exactly once in the order of decreasing 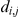. This procedure establishes a mapping (encoding) 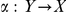. Since each tour 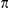 can be reached by taking 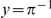, 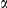 is complete. In the encoded landscape, two states 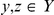 are adjacent if they differ at exactly one position (city) 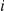.

### Maximum Cut

The last example we consider is a Spin Glass problem. Consider the set of configurations 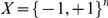 with the energy function
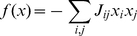
(3)for a spin configuration 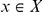. Proceeding differently from the usual Gaussian or 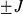 spin glass models [28], [29], we allow the coupling to be either antiferromagnetic or zero, 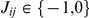. This is sufficient to create frustration and obtain hard optimization problems. Taking the negative coupling matrix 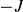 as the adjacency matrix of a graph 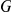, the spin glass problem is equivalent to the max-cut problem on 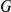, which asks to divide the node set of 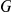 into two subsets such that a maximum number of edges runs between the two subsets [1].

The idea for an encoding works on the level of the graph 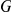, which we assume to be connected. The set 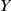 of the encoding consists of all spanning trees of 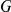. In the mapped configuration 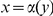, 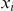 and 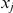 have different spin values whenever 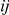 is an edge of the spanning tree 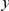. Since a spanning tree is a connected bipartite graph, this uniquely (up to 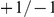 symmetry) defines the spin configuration 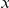. The encoding 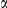 is not complete in general. Homogeneous spin configurations, for instance, are not generated by any spanning tree. Each ground state configuration 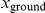, however, is certain to be represented by a spanning tree due to the following argument. Suppose there is a minimum energy configuration 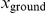 that is not generated by any spanning tree. Then the subgraph of 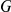 formed by all edges connecting unequal spins in 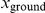 is disconnected. We choose one of the connected components, calling its node set 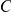. By flipping all spins in 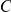, we keep all edges present for 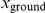. Since 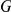 is connected, we obtain at least one additional edge from a node in 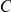 to a node outside 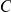. Thus we have constructed a configuration with strictly lower energy than 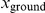, a contradiction. Two spanning trees 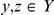 are adjacent, if 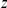 can be obtained from 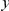 by addition of an edge 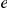 and removal of a different edge 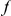.

### Enrichment

**Figure 1:**
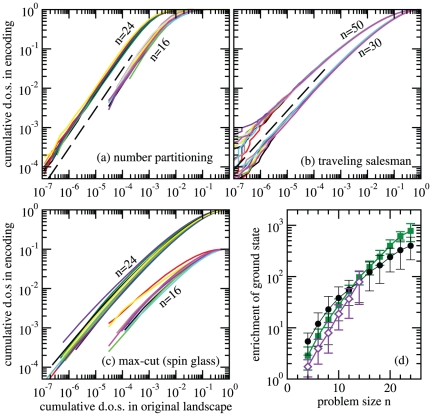
Enrichment of the density of low energy states for landscape encodings. In panels (a,b,c), a point 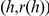 on a curve indicates a fraction 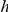 of all states have an energy not larger than a certain threshold 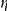 in the original landscape whereas this fraction is 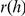 using the same energy threshold in the encoding. Panel (d) shows the average enrichment of the ground state as a function of problem size for traveling salesman (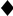), number partitioning (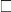), and max-cut (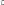). Error bars give the standard deviation over 100 independent realizations. In panels (a–c), the solid curves are for 10 random instances of each landscape and system size. The dashed lines follow 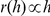 in panel (a) and 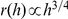 in panel (b).

We now study enrichment as well as landscape structure on these three rather different problems. To this end we consider the cumulative density of states
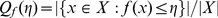
(4)in the original landscape and 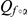 defined analogously in the encoded landscape. In order to quantify the enrichment of good solutions, we compare the fraction 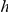 of all states with an energy not larger than a certain threshold 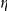 in the original landscape with the fraction 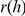 using the same threshold in the encoding. The encoding thus enriches low energy states if 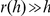 for small 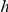. Figure 1 shows that this is the case for the three landscapes and encodings considered here. We find in fact that the density of states 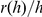 is enriched by several orders of magnitude in the encoded landscape, for all the cases considered.

Reassuringly, this trend of enrichment persists all the way to the ground state: that is, the encodings contain many more copies of the ground state than the original landscape. It appears in fact that the enrichment of ground states increases exponentially with system size. We can thus conclude that with the choice of an appropriately encoded landscape, it is easier *both* to find lower energy states from higher energy ones, and thus have more routes to travel to the ground state, as well as to *reach* the ground state itself from a low-energy neighbor, as a result of enrichment.

### Neighborhoods and neutrality

We continue the analysis of the encodings with attention to geometry and distances. A *neutral* mutation is a small change in the genotype that leaves the phenotype unaltered. In the present setting, a neutral move in the encoding is an edge 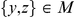 such that 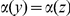. In general, the set of neutral moves is a subclass of all moves leaving the energy unchanged. An edge 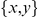 with 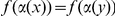 but 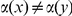 is not a neutral move in the present context. In the following, we examine the fraction of neutral moves for the encoded landscapes mentioned above.

**Figure 2:**
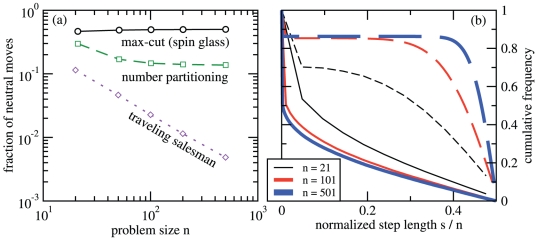
Neutrality and encoded step length. (a) The fraction of neutral neighbors as a function of problem size. (b) The cumulative distribution of the distance moved in the original landscape by a single step in the encoding. Solid curves are for the max-cut, dashed curves for the number partitioning problem, with curve thickness distinguishing values of problem size 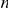. For both plots (a) and (b), data have been obtained by uniform sampling of 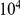 neighboring state pairs on 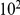 independently generated instances of each type of landscape.

Figure 2(a) shows that the fraction of neutral moves approaches a constant value when increasing the problem size of NPP and max-cut. The fraction of neutral moves in the traveling salesman problem, on the other hand, decreases as 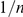 with problem size 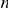. The average number of neighbors encoding the same solution grows linearly with 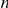, since the total number of neighbors is 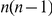 for each 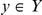 in the TSP encoding.

If a move in the encoding is non-neutral, how far does it take us on the original landscape? We define the step length of a move 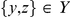 as the distance between the images of 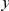 and 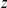 on the original landscape,
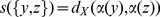
(5)using the standard metric 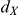 on the graph 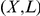. Obviously, 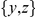 is neutral if and only if 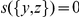. Figure 2(b) compares the cumulative distributions of step length for number partitioning and max-cut. It is intractable to get the statistics of 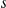 for the TSP problem for larger problem sizes since sorting by reversals, i.e., measuring distances w.r.t. to the natural move set, is a known NP-hard problem [30].

For the encoding of number partitioning, step lengths are concentrated around 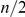. Making a non-neutral move in this encoding is therefore akin to choosing a successor state at random. For the max-cut problem, the result is qualitatively different. Step lengths are broadly distributed with most moves spanning a short distance on the original landscape. Based on this it is tempting to conclude that optimization proceeds in ‘smaller steps’ on the max-cut landscape, than in the NPP problem.

### Evolutionary dynamics

One might ask if the encoded landscape also facilitates the search dynamics, by virtue of its modified structure, and offers another avenue for optimization. For this purpose, we consider an optimization dynamics as a zero-temperature Markov chain 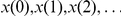. At each time step 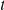, a proposal 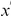 is drawn at random. If 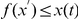, we set 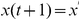, otherwise 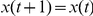. This is an Adaptive Walk (AW) when the proposal 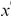 is drawn from the neighborhood of 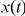. In Randomly Generate and Test (RGT), proposals are drawn from the whole set of configurations independently of the neighborhood structure. Thus a performance comparison between AW and RGT elucidates if the move set is suitably chosen for optimization. Because of the enrichment of low energy states by the encodings, it is clear that RGT performs strictly better on the encoding than on the original landscape.

**Figure 3:**
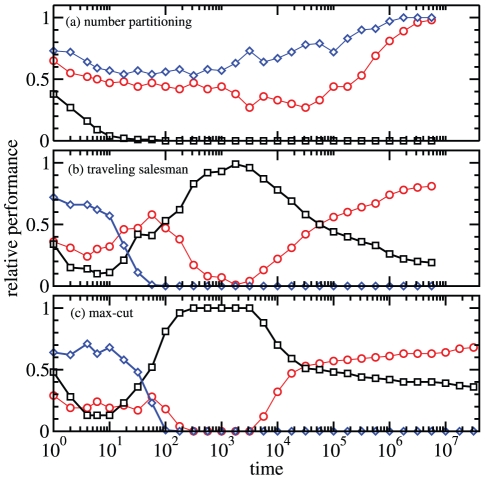
Performance comparison between three types of stochastic dynamics: adaptive walks (AW) on the original (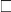) and encoded (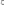) landscapes and randomly generate and test (RGT) on the encoded landscape (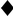). The plotted performance value is the fraction of instances for which the considered evolutionary dynamics is “leading” at time 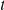, i.e. has an energy not larger than the other two types of dynamics. For each landscape, 100 random instances are used with sizes 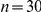 in panels (a) and (b), 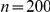 in panel (c). On each of the instances, each type of evolutionary dynamics is run once with randomly drawn initial condition 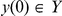 for RGT and AW in the encoded landscape. The AW on the original landscape is initialized with the mapped state 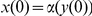. Thus all three dynamics are started at the same energy.

Adaptive walks also perform strictly better on the encoding than on the original landscape, at least in the long-time limit, cf. Figure 3. Beyond this general benefit of the encodings, the dynamics shows marked differences across the three optimization problems. In the NPP problem, RGT outperforms AW on the encoded landscape, so that enrichment alone is responsible for the increase in optimization with respect to the original landscape. In the encodings of the other two problems, AW performs better than RGT so that we can conclude that the improved structure of the encoded landscape is also an important reason for the observed increase in performance, in addition to simple enrichment. The dynamics on the max-cut landscapes (panel c) has the same qualitative behavior as that on the TSP (panel a). Although there is a transient for intermediate times where adaptive walks on the original landscape seem to be winning, the asymptotic behavior is clear: adaptive walks on the encoded landscape perform best.

### Conclusion

We have examined the role of encodings in arriving at optimal solutions to NP-complete problems: we have constructed encodings for three examples, *viz.* the NPP, Spin-Glass and TSP problems, and demonstrated that the choice of a good encoding can indeed help optimization. In the examples we have chosen, the benefits arise primarily as a result of the enrichment of low-energy solutions. A secondary effect in some but not all encodings considered here is the introduction of a high degree of neutrality. The latter enables a diffusion-like mode of search that can be much more efficient than the combination of fast hill-climbing and exponentially rare jumps from local optima. The two criteria, (1) selective enrichment of low energy states and, where possible, (2) increase of local degeneracy, can guide the construction of alternative encodings explicitly making use of *a priori* knowledge on the mathematical structure of optimization problem. The qualitative understanding of the effect of encodings on landscape structures in particular resolves apparently conflicting “design guidelines” for the construction of evolutionary algorithms.

The beneficial effects of enriching encodings immediately pose the question whether there is a generic way in which they can be constructed. The constructions for the NPP and TSP encodings suggest one rather general design principle. Suppose there is a natural way of decomposing a solution 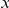 of the original problem into partial solutions. We can think of a partial solution 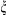 as the set of all solutions that have a particular property. In the TSP example, 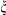 refers to a set of solutions in which a certain list 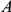 of cities appears as an uninterrupted interval. Now we choose the encoding 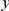 so that it has an *interpretation* as a collection 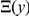 of partial solutions. A deterministic optimization heuristic can now be used to determine a good solution 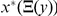. In the case of the TSP, 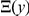 corresponds to a set of constrained tours from which we choose by a greedy solution. Alternatively, 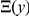 may over-specify a solution, in which case the optimization procedure would attempt to extract an optimal subset of 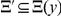 so that 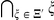 contains a valid solution 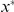. In either case, 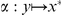 is an encoding that is likely to favour low-energy states. It is not obvious, however, that the spanning-tree encoding for max-cut can also be understood as a combination of partial solutions. It remains an important question for future research to derive necessary and sufficient conditions under which optimized combinations of partial solutions indeed guarantee that the encoding is enriching.

## Methods

### Landscapes and encoding

A finite discrete energy landscape 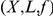 consists of a finite set of configurations 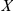 endowed with an adjacency structure 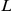 and with a function 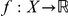 called energy, and hence 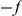 fitness. The global minima of 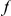 are called ground states. 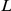 is a set of unordered tuples in 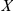, thus 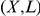 is a simple undirected graph. Let 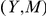 be another simple graph and consider a mapping 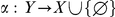, which we call an encoding of 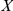. Then 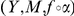 is again a landscape. (If we include states in 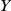 that do not encode feasible solutions we assign them infinite energy, i.e., 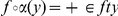 if 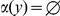.) The encoding is *complete* if 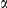 is surjective, i.e., if every 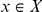 is encoded by at least one vertex of 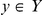. Both landscapes then describe the same optimization problem. In the language of evolutionary computation, 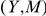 is the genotype space, while 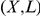 is the phenotype space corresponding to the “direct encoding” of the problem. With this notation fixed, our problem reduces to understanding the differences between the genotypic landscape 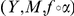 and the phenotypic landscape 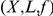 w.r.t. optimization dynamics.

### Test Instances

Random instances fox max-cut (spin glass) are generated as standard random graphs [31] with parameter 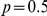: each potential edge is present or absent with equal probability, independent from other edges. Distances 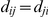 for the symmetric TSP and numbers 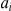 for NPP are drawn independently from the uniform distribution on the interval 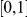.

### Enrichment factor and Density of States

The *enrichment factor* 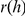 can be obtained directly from the cumulative densities of states of the two landscapes:
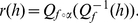
(6)This expression is a well-defined function for arguments 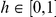 because 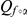 only changes value where 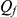 also does. For ground state energy 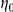, the enrichment of the ground state is 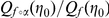.

The results in Figure 1(a–c) are obtained by sampling 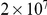 uniformly drawn states each from the original states 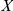 and the prepartitionings 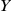 for the traveling salesman. For the two other problems, the density of states of the original landscapes is exact by complete enumeration. For the spin glass also, the density of states for 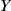 is exact from calculation based on the matrix-tree theorem. For number partitioning, 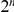 samples in 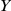 are drawn at random.

The enrichment of the ground state, Figure 1(d), is an average over 100 realizations for each problem type and size 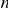. For each realization of number partitioning and max-cut, 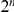 uniform samples in 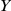 are taken; the ground state energy itself is obtained by complete enumeration of 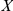. For each realization of the traveling salesman problem, 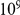 uniform samples are taken in 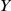; the ground state energy is computed with the Karp-Held algorithm [32].
